# Lung Acetate Levels Decline in Correlation With Increased Type 2 Allergic Markers in a House Dust Mite Allergic Mouse Model

**DOI:** 10.1002/clt2.70082

**Published:** 2025-08-04

**Authors:** Roos E. M. Verstegen, Rolf W. Sparidans, Atanaska I. Kostadinova, Johan Garssen, Gert Folkerts, Rudi W. Hendriks, Linette E. M. Willemsen

**Affiliations:** ^1^ Division of Pharmacology Faculty of Science Utrecht Institute for Pharmaceutical Sciences Utrecht University Utrecht the Netherlands; ^2^ Danone Research & Innovation Utrecht the Netherlands; ^3^ Department of Pulmonary Medicine Erasmus MC, University Medical Center Rotterdam Rotterdam the Netherlands

**Keywords:** asthma, microbiome, short chain fatty acids

## Abstract

**Aims:**

Microbial dysbiosis is an important feature in allergic asthma. Short‐chain fatty acids (SCFA) produced by the intestinal microbiome play a role in the gut‐lung axis. Little is known about how the gut SCFA levels reflect SCFA levels in other tissues and how these link to the allergic asthma inflammatory status.

**Materials and Methods:**

Male BALB/c mice were intranasally exposed to house dust mite (HDM) to induce allergic airway inflammation. Acetate, propionate, and butyrate levels were determined in caecum content, serum and lungs of control and HDM‐allergic mice using liquid chromatography‐mass spectrometry. Faecal microbiome composition was determined by DNA sequencing.

**Results:**

Mean acetate:propionate:butyrate ratios were 75:15:10 in caecum content, 98:1.5:0.5 in serum, and 38:61:1 in the lung. SCFA levels did not correlate across compartments and propionate was relatively high in the lungs. The faecal microbiome of allergic mice differed from control, with increased Desulfovibrionaceae abundance. The lung acetate proportion was lower in allergic mice compared to control. In allergic mice, declining lung acetate levels correlated with increased HDM‐specific IgE in serum and IL‐13 release by ex vivo HDM‐restimulated lung cells. Ex vivo acetate supplementation did not inhibit HDM‐restimulated lung cell IL‐13 production, while butyrate and propionate did.

**Conclusions:**

Overall, HDM‐driven murine allergic airway inflammation induced changes in the faecal microbiome and reduced acetate in serum and lung tissue. Hereby, lung acetate levels correlated negatively with sensitisation and type‐2 inflammation, but acetate itself did not suppress ex vivo HDM‐induced cytokine release. Our findings provide new insights on the systemic effects of HDM‐allergic inflammation along the gut‐lung axis.

## 1 Introduction

Almost 300 million people worldwide suffered from asthma in 2019 [1]. Microbial dysbiosis, both in the gut and the lung, can play a role in the high asthma incidence [2]. The intake of dietary fibres, and the resulting metabolites of their fermentation in the gut, including short‐chain fatty acids (SCFA), have been associated with a healthy gut and lower prevalence of chronic noncommunicable immune and metabolic disorders [3]. The major produced SCFAs are acetate, propionate and butyrate [4]. It is becoming increasingly clear that by acting both locally, and systemically via absorption by the gut and after passing the liver, SCFAs can beneficially affect the disease burden of immune‐related disorders, such as allergic asthma [5, 6].

Although the importance of SCFAs is well renowned, limited information exists on the actual levels of SCFAs in different body compartments. Studies mainly report on SCFA levels in the caecum and/or faeces in pre‐clinical settings or faeces in clinical trials. The ratio of acetate, propionate, butyrate in adults was reported as 57:22:21, with a total concentration of 80 mM in the descending colon. The proportion of acetate increases throughout the route to the portal blood, hepatic blood and peripheral blood, which ratios were described as 71:21:8, 81:12:7 and 91:5:4, respectively. Simultaneously, SCFA concentrations drop in these compartments from 375 to 140 to 79 μM respectively [7]. Less is known about SCFA levels in the lung, and how these relate to SCFA levels in other compartments that are typically sampled, including faeces, caecum content or blood.

Allergic asthma is a chronic disease that can be induced by many allergens, including house‐dust‐mite (HDM), causing sensitisation that results in eosinophilic airway inflammation and allergen‐specific IgE production [8]. As SCFA have immune‐modulatory properties, some clinical studies were performed aimed at increasing SCFA levels in allergic patients, for example by dietary fibre interventions [9]. Although some human studies considering the association between SCFAs and allergic diseases exist [10], the effects of allergic airway inflammation on SCFA profiles, especially for body compartments beyond the gut, are largely unknown. Therefore, we studied SCFA profiles in different body compartments of HDM‐allergic mice, using sham mice as control, and determined whether these correspond to the type 2 inflammatory status in HDM‐allergic mice. Furthermore, we investigated effects of HDM‐driven allergic airway inflammation on the faecal microbiome.

## 2 Materials and Methods

### 2.1 Animals

In two independent experiments, male BALB/cAnNCrl mice (Charles River, Germany), aged 7 weeks, were co‐housed in individually‐ventilated cages under standardised conditions. The housing environment featured a 12 h/12 h light/dark cycle, controlled temperature (21 ± 2°C), relative humidity maintained at 50%–55%, woodchip bedding, wood‐curl nesting material, a plastic shelter and a plastic tube. Mice were fed a semi‐synthetic AIN93G diet without glucose/lactose (ssniff‐Spezialdiëten GMBH, Germany) *ad libitum*, to stay in line with previous work in our group [11, 12, 13], and had access to sterile water. Mice (first experiment *N* = 18, second experiment *N* = 12) were randomly allocated to experimental groups after arrival. Researchers were blinded during the study until analysis results were finished. The experiments adhered to the institutional Guidelines of the Ethical Committee on the Use of Laboratory Animals of Utrecht University (AVD1080020198826). Animal procedures were approved by the local Animal Welfare Body under an ethical licence provided by the national competent authority (Centrale Commissie Dierproeven, CCD), securing full compliance to the European Directive 2010/63/EU for the use of animals for scientific purposes.

### 2.2 Animal Procedures

An allergic airway inflammation model was performed during the light/inactive phase of the mice, according to previous work [11], but with the following adaptations: after 2 weeks of acclimatisation, isoflurane anaesthetised mice were sensitised intranasally (i.n.) with 40 μL PBS in absence (sham control group, *N* = 10) or presence (allergic group, *N* = 20) of 5 μg HDM (CITEQ, 02.01.86, Groningen, the Netherlands). Daily i.n. challenges followed on days 7–11 with PBS in absence or presence of 15 μg HDM. Mouse faeces was collected during an individual weighing moment in an open box on day 11. Anaesthetics on day 14 consisted of two 150 μL i.p. injections of a solution containing 4.92 mg ketamine and 0.033 mg Dexdormitor, and were followed by terminal cardiac puncture.

### 2.3 Caecum Content

Caecum content was weighted and diluted in 1 mL ice‐cold PBS. After addition of 1.0 mm glass beads (BioSpec Products), the samples were vortexed 1.5 min, and centrifuged for 10 min at 16,873 × g (4°C). Supernatant was stored at −20°C until further analysis.

### 2.4 Serum

Blood was collected in MiniCollect TUBES (Greiner) and was left at room temperature for min. 30 min to coagulate. Samples were then centrifuged at 16,873 × g (room temperature) for 10 min. Serum was collected and stored at −20°C until further use. Blood of two mice of the same experimental group in one of the experiments was pooled due to low blood yield.

### 2.5 Lung Homogenates

500 μL PBS solution containing 1% (v/v) Triton X100 (Sigma‐Aldrich) and protease inhibitor tablets (1 per 10 mL) (Complete Mini, Roche Diagnostics) was added to weighted lung samples in a 2 mL homogenisation tube (CKMix tubes, Bertin). After homogenisation using the Precellys 24 tissue homogeniser (Bertin), homogenates were centrifuged for 10 min at 16,873 × g (4°C). Supernatants were collected and stored at −20°C until further use.

### 2.6 Short‐Chain Fatty Acid Determination Using LC‐MS/MS

Acetate, propionate and butyrate concentrations were quantified in 10 μL caecum content homogenate supernatant, serum and lung homogenate supernatant by means of liquid chromatography‐tandem mass spectrometry (LC‐MS/MS) using the Shimadzu Nexera X2 chromatographic system (Kyoto, Japan) and the AB‐SCIEX QTRAP 5500 triple quadrupole mass spectrometer (Ontario, Canada). Internal standards included acetic acid‐d_4_ (50 μM) (Thermo‐Scientific), propionic acid‐d_3_ (20 μM) (Toronto Research Chemicals) and butyric acid‐d_7_ (10 μM) (Cayman chemical). See Supporting Information S1 for more details.

SCFA concentrations of caecum content and lung were corrected for the initial tissue weight, by adjusting the values of the SCFA concentration to the amount of mol SCFA per gram of tissue. To allow the combination of two independent experiments, average SCFA concentrations in the control group, or in the allergic group in case only data from the allergic group were used, of each experiment were set to 100% and individual values of each mice within the experiment were expressed relatively to this normalised value.

### 2.7 Faecal Microbiome Analyses

Approximately 70 mg of faeces was collected from all mice on day 11. According to standard protocol, *N* = 5 (minimum) sample analyses provide sufficient power to perform microbiome analysis. Overall, in both studies samples of randomly selected mice from each experimental group (total control *N* = 10, total allergic *N* = 11) were sent to BGI Genomics (Hong Kong) for microbiome sequencing. Details of DNA extraction, library preparation, DNA amplification, PCR enrichment and sequencing, as written down by BGI, can be found in the Supporting Information S1.

Microbiome composition was analysed on family level. Differences between control and HDM‐allergic mice were tested per bacterial family.

### 2.8 Eosinophil Influx in Bronchoalveolar Lavage Fluid (BALF)

Cells in BALF were collected as described previously [11]. Cells were counted using a Bürker‐Türk chamber and identified by performing flow cytometry. See Supporting Information S1 for details.

### 2.9 Ex Vivo HDM Restimulation of Lung Cells

Lung tissue was processed to a single cell suspension and restimulated with HDM as described previously [11], except for a different HDM dose (25 μg/mL, Citeq) and a longer incubation time (6 days). Additionally, single cell suspensions from allergic mice were restimulated with HDM in presence of 1 mM of either acetate, propionate or butyrate (Sigma‐Aldrich).

### 2.10 Antibody and Cytokine Determination Using ELISA

HDM‐specific IgE in serum and IL‐13 in supernatant of restimulated lung cells were measured using ELISA. IL‐13 (Invitrogen) was measured according to the manufacturer's protocol. Due to a practical error, IL‐13 concentration upon lung HDM restimulation was not measured for one allergic mouse. For HDM‐IgE, an in house protocol was used [14]. An adaptation from this protocol is the coating concentration of HDM (10 μg/mL). Due to absence of a standard, serum HDM‐IgE is expressed as optical density (OD) value, which was the read‐out value of this measurement.

### 2.11 Statistics

Sample size calculations were performed using G*Power 3.1.9.2 software. An ANOVA (fixed effects, omnibus, one‐way) test was performed with a priori power analysis. Input from previous experiments in our group were used to determine the effect size. One mouse from Experiment 2 was excluded from the allergic group due to failure to develop any increase for one of the inflammatory markers.

Statistical analyses were conducted using GraphPad Prism software (version 10.4.1). All data were checked for Gaussian distribution and transformed when necessary. For correlation testing, a Pearson correlation coefficient was calculated. A nonparametric Spearman was used in case data for correlation were not normally distributed after transformation.

Comparisons between two variables were performed using unpaired t‐tests. If data did not adhere to the conditions for the test, a non‐parametric Mann‐Whitney test or unpaired *t*‐test with Welch correction was performed. Comparisons between multiple variables were tested using one‐way ANOVA. If data did not adhere to the test conditions, Brown‐Forsythe and Welch ANOVA were performed with Dunnett's T3 multiple comparisons test. For all analyses, two‐tailed *p*‐values were generated. *p* < 0.05 was considered significant.

## 3 Results

### 3.1 Short‐Chain Fatty Acid Composition Varies Across Mouse Tissue Compartments

**FIGURE 1:**
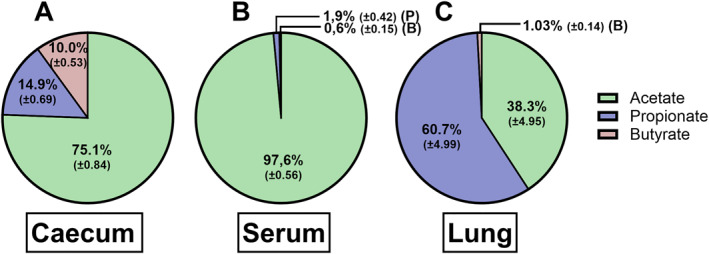
Ratios of acetate, propionate and butyrate in caecum content (A), serum (B) and lung (C). Results of two independent studies are included. Results are shown as mean ± SEM. *N* = 10.

SCFA levels were determined in the caecum content, in serum, and in lungs of control mice by LC‐MS/MS (Table 1). To allow comparison between compartments, we determined the mean ratios of acetate:propionate:butyrate, which were approximately 75:15:10 in the caecum content (Figure 1A), 97.5:2:0.5 in serum (Figure 1B) and 38:61:1 in the lung (Figure 1C).

**TABLE 1:** Short‐chain fatty levels in caecum content, serum and lung homogenate in sham mice.

	Caecum content (nmol/g tissue)	Serum (μM)	Lung (nmol/g tissue)
Total SCFA	244 (± 30)	427 (± 92)	3.313 (± 0.679)
Acetate	184 (± 23)	420 (± 92)	1.351 (± 0.316)
Propionate	35 (± 4)	5 ± (0.3)	1.934 (± 0.386)
Butyrate	24 (± 3)	1 ± (0.1)	0.028 (± 0.004)

*Note:* Sham mouse were intranasally exposed to PBS. Results of two independent studies are included. Total SCFA = sum of acetate, propionate and butyrate. Mean ± SEM. *N* = 10.

### 3.2 Short‐Chain Fatty Acid Levels Do Not Positively Correlate Between Compartments

**FIGURE 2:**
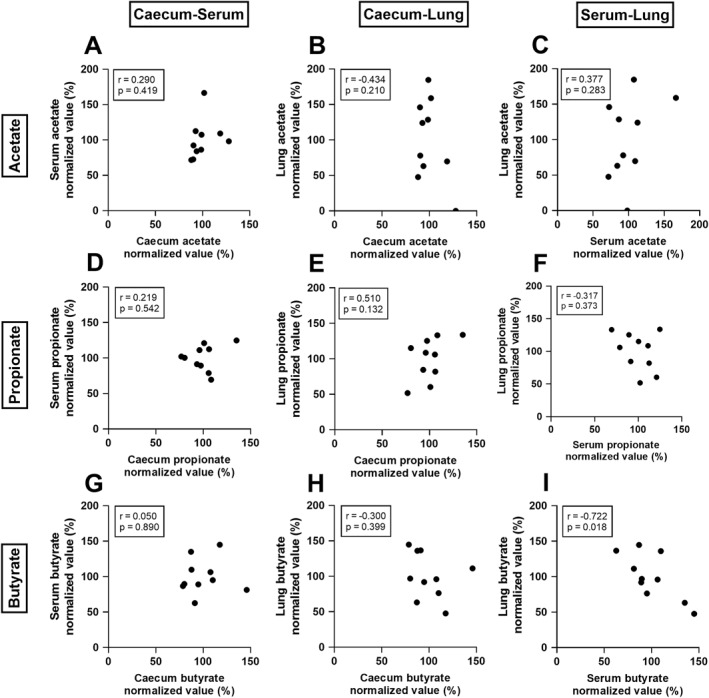
Correlations of short‐chain fatty acid (SCFA) levels between caecum‐serum, caecum‐lung and serum‐lung in control mice. (A–C) Correlations of acetate (D–F) propionate or (G–I) butyrate between the three compartments. SCFA levels are presented as percentages, whereby the average values of the individual experiments were set to 100%. Data was transformed when not normally distributed. Correlations were determined by computing Pearson correlation coefficients. Results of two independent studies are included. Each dot represents one control mouse. *N* = 10.

To investigate if intestinal SCFA levels, determined in the caecal content, were reflected in the serum or lungs within control mice, these levels were correlated (Figure 2). For acetate and propionate, we observed no significant correlations between the three compartments studied. Butyrate levels correlated negatively between serum and lung (Figure 2I), but not in the other two tissue comparisons.

### 3.3 HDM Allergic Mice Show Changes in Faecal Microbial Composition, Including Increased Desulfovibrionaceae Abundance

**FIGURE 3:**
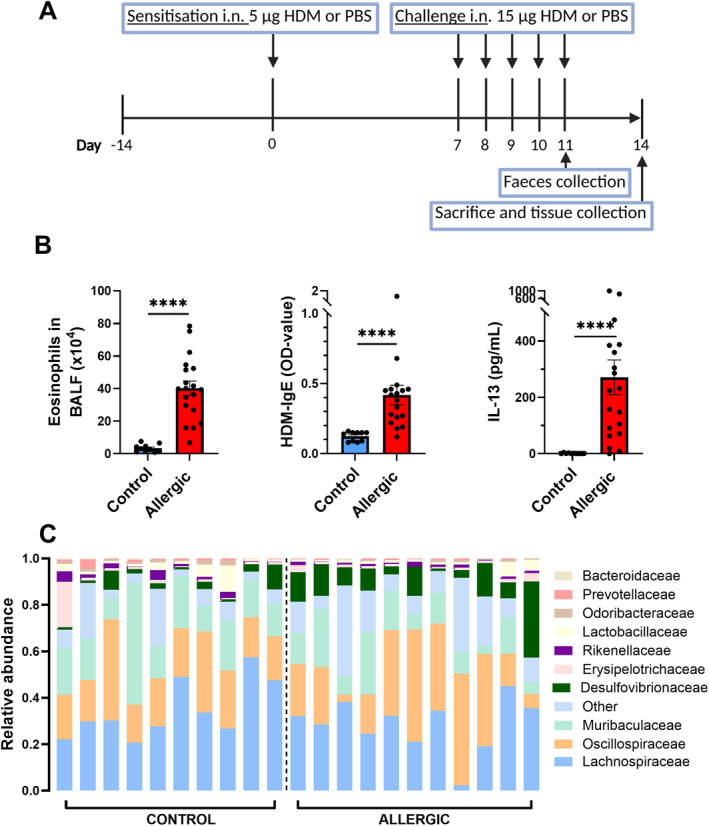
The effects of house dust mite (HDM)‐induced allergic airway inflammation on the faecal microbiome in mice. (A) Schematic overview of the HDM‐induced allergic airway inflammation model. (B) Confirmation of allergic airway inflammation in allergic versus control mice, by eosinophil influx in the bronchoalveolar lavage fluid (BALF), HDM‐specific IgE levels in serum and IL‐13 production by lung cells upon ex vivo HDM‐restimulation. Data were analysed by either a Welch's test or Mann‐Whitney test and are shown as mean ± SEM (****p < 0.0001). Results of two independent studies are included. *N* = 10 for control, *N* = 18–19 for allergic. (C) Top 10 bacterial families per experimental group. Residual families were labelled as other. *N* = 10 for control, *N* = 11 for allergic.

Next, we induced HDM‐driven allergic airway inflammation in mice by intranasal exposure to HDM (Figure 3A). The airway inflammation was characterised by increased number of eosinophils in the BALF, the presence of HDM‐specific IgE in serum and allergen‐specific IL‐13 release by lung cell suspensions after 6 days of ex vivo HDM restimulation, compared to control mice (Figure 3B).

Analysis of bacterial DNA from faecal samples showed that the abundance of most bacterial families was similar in control and allergic mice (Figure 3C). However, we observed a significant increase in relative abundance of *Desulfovibrionaceae* (*p* = 0.0082) and a decrease of *Rikenellaceae* (*p* = 0.0133) in HDM‐allergic mice. A decreasing trend of the *Muribaculaceae* (*p* = 0.0775) and *Prevotellaceae* (*p* = 0.0848) was observed in allergic mice compared to the control group.

### 3.4 HDM‐Allergic Mice Show Reduced Serum Acetate Levels and Reduced Acetate Proportions in Lung Homogenates

**FIGURE 4:**
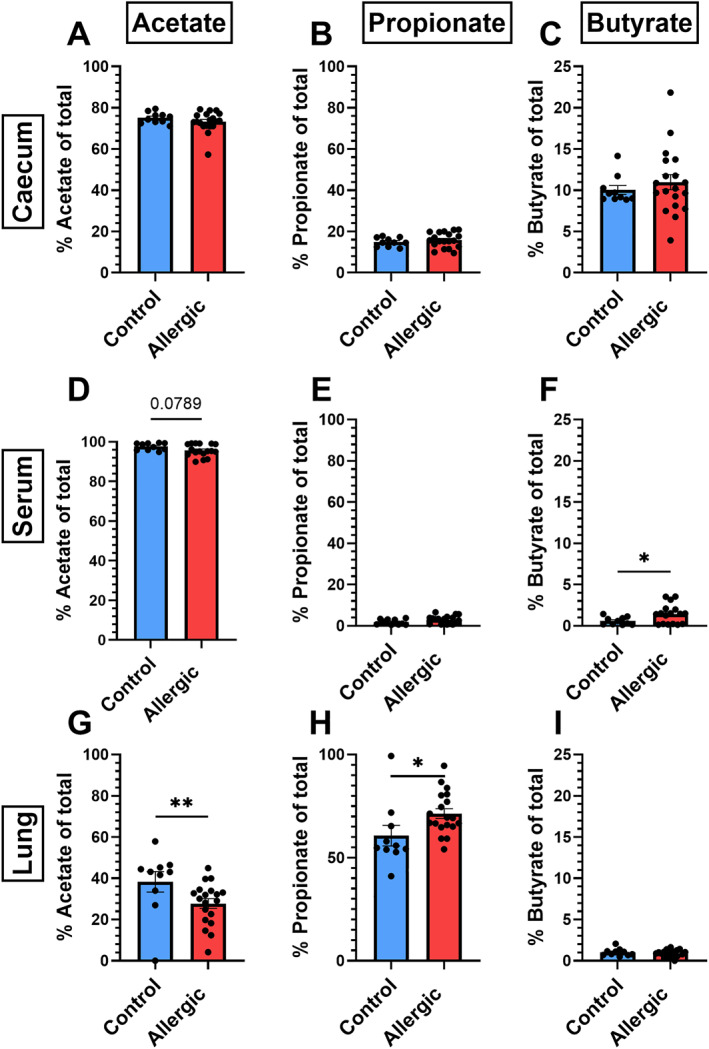
Short‐chain fatty acid (SCFA) proportions compared between control and house dust mite (HDM)‐allergic mice in caecum content, serum and lung. For each mouse the percentage of acetate, propionate and butyrate per compartment was determined. Results of two independent studies are shown together. Data were analysed by an unpaired *t*‐test. Data were transformed if needed, or analysed using a non‐parametric Mann‐Whitney test in case transformation did not normalise the data. *N* = 10 for control, *N* = 19 for allergic. Data are shown as mean ± SEM (*p < 0.05, **p < 0.01).

Next, we compared SCFA ratios in the different body compartments between control mice and HDM‐allergic mice. In the caecum, none of the SCFA ratios significantly differed between control and allergic mice regarding levels (Supporting Information S1: Figure S2A–C) and proportions (Figure 4A–C). In serum, acetate levels significantly declined in HDM‐allergic mice (Supporting Information S1: Figure 2D), which was also reflected in a trend of decreased acetate proportion (Figure 4D), and accompanied by an increased proportion of butyrate (Figure 4F). In the lung, the acetate proportion significantly decreased in HDM‐allergic mice (Figure 4G), while the propionate proportion increases (Figure 4H). Based on the absolute values, this effect is attributed to a decreased presence of acetate in the lung, while propionate levels stay similar (Supporting Information S1: Figure 2GH).

### 3.5 Decline of Lung Acetate Correlates With Increased Allergic Airway Inflammation Parameters

**FIGURE 5:**
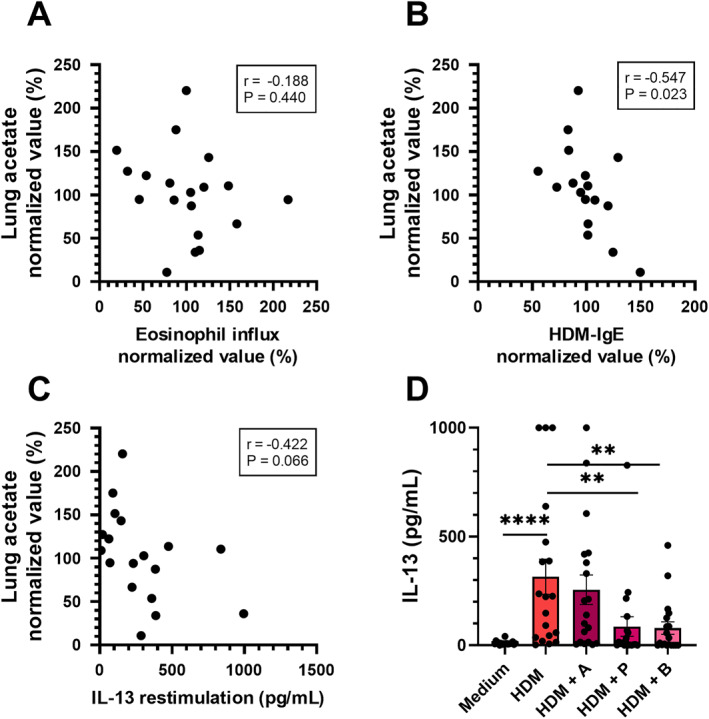
Correlations of inflammatory outcomes in house dust mite (HDM)‐allergic mice with lung acetate levels and effects of ex vivo short‐chain fatty acid (SCFA) treatment on HDM‐restimulated lung cells. (A) Eosinophilic influx in bronchoalveolar lavage fluid (BALF) (normalised*) versus acetate level in the lung (normalised*). (B) HDM‐IgE in serum (normalised*) versus acetate level in the lung (normalised*). (C) IL‐13 release from ex vivo HDM‐restimulated lung cells versus acetate levels in the lung (normalised*). (D) IL‐13 release from ex vivo HDM‐restimulated lung cells in the absence or presence of 1 mM acetate, propionate or butyrate. Each dot represents one HDM‐exposed mouse. Correlation was determined by computing Pearson correlation coefficients. HDM‐specific response was determined by Mann‐Whitney test. Difference between groups was determined by Brown‐Forsythe and Welch ANOVA tests, with Dunnett T3 multiple comparisons test. *N* = 18–19. Results of two independent experiments are shown in the graphs. *Normalised values; average values of the individual experiments were set to 100%.

Although lung acetate levels did not correlate with eosinophilic cell influx in the BALF of HDM‐allergic mice (Figure 5A), they did negatively correlate with inflammation markers HDM‐IgE in the serum (Figure 5B) and IL‐13, released upon ex vivo HDM‐restimulation of lung cell suspension (Figure 5C), or showed a negative correlation trend.

Ex vivo acetate exposure during HDM‐restimulated lung cell cultures of HDM‐allergic mice did not prevent allergen‐specific IL‐13 release by lung cell suspensions (Figure 5D). In contrast, propionate and butyrate treatment did prevent IL‐13 release. These findings suggest that the observed negative correlation of lung acetate and IL‐13 in HDM‐allergic mice (Figure 5C) cannot be explained by a direct effect of acetate on the IL‐13 production capacity of lung T cells.

## 4 Discussion

SCFAs are fermentation end‐products that can affect systemic immunity directly and indirectly, for example by affecting bone marrow precursor cells [5]. Although there is consensus about the protective role SCFAs can play in (allergic) inflammation, knowledge about systemic SCFA profiles in health and (allergic) disease is sparse. In this study, we aimed to gain insight in the distribution of SCFAs in caecum content, serum and lungs of mice, and in the correlation of SCFA levels between these compartments. Furthermore, we aimed to elucidate the effect of HDM‐induced allergic airway inflammation on this SCFA distribution and whether this can be linked to the type 2 inflammation status of HDM‐allergic mice. We found that the mean acetate:propionate:butyrate ratios were 75:15:10 in caecum content, 98:1.5:0.5 in serum and 38:61:1 in the lungs. In individual mice, SCFA levels did not positively correlate across compartments and propionate was relatively high in the lungs. The faecal microbiome composition of allergic mice differed from control, with increased *Desulfovibrionaceae* abundance. The serum acetate level and lung acetate proportion was lower in allergic mice compared to control. In allergic mice, declining lung acetate levels correlated with increased HDM‐specific IgE in serum and ex vivo IL‐13 release by HDM‐restimulated lung cells. The latter was not prevented using ex vivo acetate treatment during HDM‐exposure.

First, we determined SCFA levels in the caecum content, serum and lungs of control mice. The acetate, propionate and butyrate proportions in the caecum and serum corresponded with earlier findings in the caecum of mice on the same diet [15, 16], and human peripheral blood [7]. The higher acetate proportion in the blood compared to the caecum can be explained by the relatively high initial concentration of acetate in the gut and the fate of propionate and butyrate. Butyrate is predominantly used as energy source by colonocytes [17], while propionate is metabolised by the liver. This leaves low levels to enter the systemic circulation [7]. In contrast, acetate is expected to enter the circulation. Absolute caecum content concentrations are difficult to compare between studies, due to differences in methods. For example, studies used different mice strains or other diets [18] or the amount of starting material is unclear [16]. Total SCFA concentrations in serum are slightly lower in our study, though in the same order of magnitude as previously reported in mice [19].

Unexpectedly, we observed that SCFA ratios in the lungs were very different from those in caecum and serum. Not acetate, but propionate was the predominant SCFA in the lung. Additionally, an unexpected negative correlation between serum and lung butyrate was observed. However, butyrate levels were relatively small compared to acetate and propionate levels in the lung, and may fuel immunometabolism in the tissue affecting steady state levels [20]. Preclinical studies to compare these findings, concerning SCFA levels in the lung, are lacking. Trompette et al. [19] reported that SCFAs in lung tissue of HDM‐exposed mice were below detection limit, which was 500 μM when using HPLC. The LC‐MS/MS method we used is more sensitive, and can quantify concentrations as low as 0.4 μM. Studies in human lung sections of cancer patients [21] and BALF of Cystic Fibrosis (CF) patients [22] revealed ratios of 80:18:2 and 91:7:2, respectively. This does not correspond to our findings in mice, albeit these were patients with different diseased conditions [17].

Based on our finding that the propionate proportion is much higher in the lung (60.7%) than in serum (1.8%), we hypothesise that propionate may be produced locally. SCFA production is not limited to the gut, as also extra‐intestinal sides have a local microbiome, such as the skin, the female genital tract [5], and the lung [23], which was for example shown in the context of CF [22]. In order to explain our finding that propionate is the dominant SCFA in the murine lung, lung microbiome analyses can be considered in future studies.

The gut microbiome, the primary producer of SCFAs, might play an important role in the development and progression of asthma. Dysbiosis, characterised by the increased abundance of harmful bacterial genera, and the decreased abundance of beneficial genera, increases the risk of asthma [24]. We investigated the microbiome and SCFA profiles in HDM‐exposed mice that developed HDM‐induced allergic airway inflammation, and compared them to the profiles of control mice.

In an ovalbumin (OVA)‐induced asthma study with BALB/c mice increased levels of *Desulfovibrionaceae* were found, similar to our experiments [25]. As this experiment involved i.p. injections with a strong adjuvant, it is conceivable that the use of an adjuvant may be accompanied by microbiome changes. In our experiments, mice were exposed to HDM i.n. However, during i.n. HDM‐exposure, some HDM‐solution might ‘leak’ to the digestive tract. There is so far no described link between *Desulfovibrionaceae* and human asthma. Nevertheless, this bacterial family is linked to many other diseases, including Parkinson's disease, inflammatory bowel disease and colorectal cancer [26]. In vitro, these bacteria are associated with induced production of pro‐inflammatory cytokines by endothelial and intestinal epithelial cells and increased permeability of the intestinal epithelial barrier [26]. These pro‐inflammatory features may affect asthma development as well. In line with our results, *Rikenellaceae* were reduced in stool samples of 1 year old children with asthma and/or infant exposed to antibiotics [27]. *Muribaculaceae* is a bacterial family present in murine faeces, but rarely in human samples [28]. They can produce SCFA, and their abundance is decreased in multiple disease models, including inflammatory bowel disease, obesity and type 2 diabetes. Therefore, a higher abundance of this family is usually considered beneficial. Other studies, in which mice were exposed to HDM or OVA, showed that allergic mice had lower levels of *Muribaculaceae* compared to control mice [29, 30]. The role of *Prevotellaceae* in the gut depends on specific factors, including the strain [31]. *Prevotella copri* is positively associated with several allergic diseases [32, 33, 34, 35], but negative immune associations with *Prevotellaceae* have also been reported [36, 37].

The microbiome of control and allergic mice differed in the relative abundance of specific bacterial families. Nevertheless, the caecal acetate, propionate and butyrate levels and ratios did not differ between control and allergic mice. This indicates that, although relative abundances of bacteria changed, functionally the microbiome was not altered consistently in allergic mice regarding SCFAs in caecal content.

In serum we did observe differences in SCFA levels between control and allergic mice, since acetate levels decreased in HDM‐allergic mice. However, the SCFA ratio was not different, probably due to the high acetate abundance in the serum. In human studies, decreased serum and or faecal acetate levels, prenatally or at early infancy, were associated with childhood wheeze and/or asthma development [10]. SCFAs are known to be transported across the colonic epithelium mainly by specific carriers, but also by SCFA/HCO_3_^‐^ exchange and nonionic diffusion [38, 39]. Changes in the microbiome might affect the expression of SCFA transporters and thus SCFA uptake across the colonic epithelium [40]. It is conceivable that the expression or function of acetate transporters in the gut of allergic mice were altered. Human studies, mainly focus on faecal SCFAs measurements, it may be considered to also aim to determine serum SCFA concentrations, as a reflection of the SCFA status in allergic asthma.

In the lung homogenates, lower acetate levels were found in allergic mice compared to control. The acetate proportion was significantly lower in lungs of allergic mice as well. Not many studies have analysed SCFA levels in the lung. One study showed that total SCFA levels in the sputum of cystic fibrosis patients tended to be lower in patients with pulmonary exacerbations compared to stable patients [41]. SCFA can be used as energy source by cells via the process of fatty acid oxidation [42]. Some immune cells, like M2 macrophages and memory T cells, rely mostly on oxidative phosphorylation and fatty acid oxidation in their energy metabolism [43]. In cancer, acetate utilisation by cells as their energy source is increased, due to changes in transporters and metabolic enzymes in the cells facilitating acetyl‐CoA formation out of exogenous acetate [42]. Possibly, a similar mechanism underlies increased acetate usage by immune cells in the bloodstream and in the lung, leading to decreased acetate levels and/or proportions.

SCFAs can dampen transcription of the sterile germline transcript (εGLT) in lymphoid tissues, including the mediastinal lymph nodes. εGLT transcription is an indicator of IgE isotype switching [44]. Regarding the allergen‐induced IL‐13 production, most likely by Th2, Tc2 cells and ILC2s [45], studies describe propionate and butyrate, potent HDAC inhibitors, can suppress type 2 activation. However, this does not apply to acetate [9]. In general, not many studies have associated SCFA levels of individuals with inflammatory markers. In stable asthma patients, researchers demonstrated lower sputum butyrate levels to be associated with higher mucus plug scores [46]. In the current study, serum butyrate levels did tend to correlate negatively with HDM‐IgE levels, but butyrate increased in serum of HDM‐allergic mice compared to control.

As lung acetate levels negatively correlated with inflammation markers, we tested if acetate could suppress type 2 activation of HDM‐restimulated lung cells. Acetate did not prevent HDM‐induced IL‐13 production, while propionate and butyrate did. Propionate and butyrate are potent HDAC inhibitors, while acetate is only known to be one to a lesser extent [5]. Since acetate did not directly affect IL‐13 production capacity of lung T‐cells, it may indirectly play a role in allergy development. Alternatively, as indicated before, it may have been used as energy source during allergic lung inflammation or may have acted to suppress IL13 in another dose or at another time‐point after in vivo HDM challenge. Future studies should further clarify the association between low lung acetate concentrations and type 2 allergic inflammation in HDM mice. The intestines of mice and humans are anatomically, histologically and physiologically quite similar. However, it must be considered that substantial differences exist, including the metabolic rate and diet affecting the microbial composition, which even differs between mouse strains [47]. Therefore, it is warranted to also translate these findings to the human situation.

In conclusion, lung acetate levels decline in a mouse model of house dust mite allergy, and correlate negatively with type 2 allergic markers. The different compartments, caecum, serum and lung, did not correlate regarding SCFA levels, underlining the importance of local SCFA measurements in future studies.

## Author Contributions

Conceptualisation by R.E.M.V., A.I.K, G.F., R.W.H. and L.E.M.W. Funding acquisition by G.F., R.W.H. and L.E.M.W. Experiments performed by R.E.M.V. Sample analysis by R.E.M.V. and R.W.S. Data interpretation by R.E.M.V., A.I.K, G.F., R.W.H. and L.E.M.W. Writing by R.E.M.V. Review and editing by R.W.S., A.I.K, G.F., J.G., R.W.H. and L.E.M.W. All authors have read and agreed to the published version of the manuscript.

## Conflicts of Interest

The authors declare no conflicts of interest.

## Supporting information

Supporting Information S1

## Data Availability

The data that support the findings of this study are available from the corresponding author upon reasonable request.
