# Supplementary material for: Lung Acetate Levels Decline in Correlation With Increased Type 2 Allergic Markers in a House Dust Mite Allergic Mouse Model
Source: Clin Transl Allergy. 2025 Aug 4;15(8):e70082. doi: 10.1002/clt2.70082 (PMC12321596; doi:10.1002/clt2.70082)
Supplement: Supplementary file 1 — Supporting Information S1 [file CLT2-15-e70082-s001.docx]

**METHODS**

**Eosinophils in bronchoalveolar lavage fluid**

Lungs were lavaged, starting with 1 mL pyrogen free saline (0.9% NaCl, 37°C) + protease inhibitor (Complete Mini, Roche). This was followed by 3 lavages of 1 mL 0.9% NaCl (37°C). Lavages were pooled and centrifuged (400 g, 5 min) to collect cells. A Bürker-Türk chamber was used to count the total number of cells in the BALF (100x magnification). Eosinophil numbers were determined by performing flow cytometry with the collected BALF cells based on a paper published by van Rijt et al^40^. In short, 60 µL solution containing BALF cells of each sample was plated and centrifuged (5 min, 4°C). Cells were then threated with Fixable Viability dye for 30 min, block buffer (1%BSA, 1% CD16/CD32 block solution) for 10 min, and specific conjugated antibodies against CD3e-Pe Cy7, MHCII-FITC, CD11c-APC, B220-Pe Cy7 (Thermo Fisher Scientific) and CCR3-PE (BioLegend) were added. Necessary washing steps were performed in between. The FACS Canto II (BD) was used for flow cytometry, followed by analysis using FlowLogic Software (Inivai Technologies). Eosinophils were gated as low FSC - CD3e/B220 negative, CCR3 positive cells (Suppl.Fig.1).


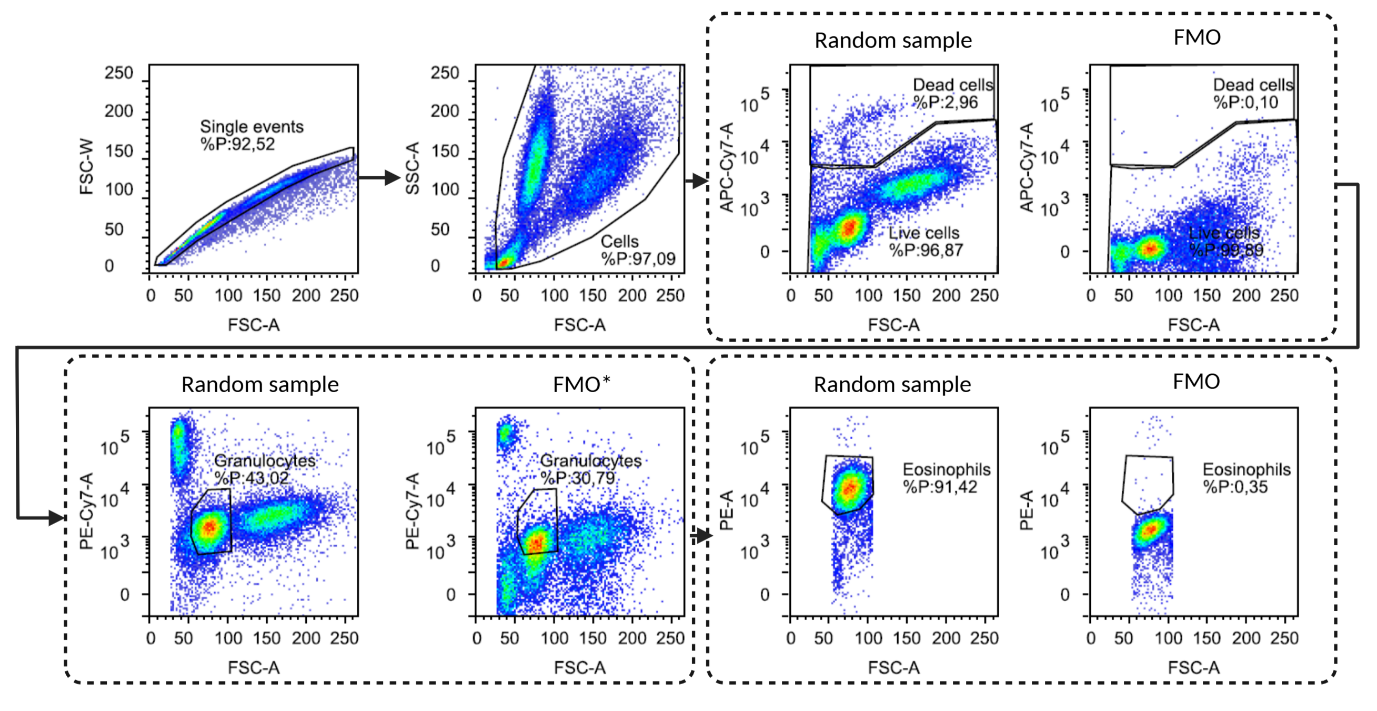


**Supplemental figure 1. Gating strategy to identify eosinophils in bronchoalveolar lavage fluid.** Upper graphs show the general gating for live cells. Granulocytes were defined as CD3e/B220 negative cells with small forward scatter. Eosinophils were defined in the granulocytes gate as CCR3 positive cells. A representative sample and corresponding FMO controls are displayed. *Due to a practical error the CD3e antibody was added to the FMO control, making it not usable for this study. Gating was confirmed by previous experiments.

**Short-chain fatty acid determination using LC-MS/MS**

***Sample and standards preparation***

Supernatant of caecum content homogenates, serum samples and supernatant of lung homogenates were analyzed. Caecum content homogenate samples were diluted in 50% (v/v) acetonitrile to fit the range of the measurements. This was not needed for serum and lung samples. 10 µL (diluted) sample and 20 µL internal standards dilution in acetonitrile were added to a polypropylene 96-wells plate with conical bottom. After closing with a silicone mat, the plate was vortex mixed vigorously for several seconds and afterwards centrifuged for 5 min at 2643 × g. Next, 20 µl of the supernatant was pipetted into a polypropylene 96-deep well plate and 10 µl of 0.2 M 3-nitrophenylhydrazine hydrochloride (3-NPH) (Sigma-Aldrich) solution in 50% acetonitrile and of 10 µl 0.12M -ethyl-3-(3-dimethylaminopropyl) carbodiimide (EDC)-hydrochloride(Carl Roth) in a mixture of pyridine/acetonitrile/water (0.5/49.5/49.5% (v/v/v)) were added. Then, the plate was closed again with the mat and gently mixed. After this, the plate was incubated in a water bath of 40°C for 30 minutes, after which the plate was put on ice. Finally, samples were diluted with 200 µL water and stored at 4°C until analysis.

Standard samples were prepared in 50% (v/v) acetonitrile in the ranges 20-5000 µM (acetate), 1-250 µM (propionate) and 0.4-100 µM (butyrate)(Sigma-Aldrich). Quality control samples were prepared in 50% (v\v) acetonitrile at 4000; 400; 40 µM (acetate), 200;20;2 µM (propionate)and 80;8;0.8 µM (butyrate). Internal standards of acetic acid-d_4_ (50 µM)(Thermo-Scientific), propionic acid-d_3_ (20 µM)(Toronto Research Chemicals)and butyric acid-d_7_ (10 -µM)(Cayman chemical) were prepared in acetonitrile. All these solutions were stored at 4 °C until analysis.

***Analytical instruments***

For separation we used the Shimadzu Nexera X2 chromatographic system (Kyoto, Japan). This contained two LC30-AD pumps, a Sil30-ACmp autosampler, DGU-250 AR degasser and a CTO-20 AC column oven. For detection we used the AB-SCIEX QTRAP® 5500 triple quadrupole mass spectrometer (Ontario, Canada), containing a Turbo Ion™ TurboIonSpray® probe and inlet valve. Analyst 1.6.2 software (Sciex) was used for data collection and instrument control. LC-MS/MS data were processed using MultiQuant 3.0.1 software (Sciex).

***LC-MS/MS conditions***

3 µL sample was injected on the VisionHT C18-P (50 x 2.0mm, 1.5 µm) with a VisionHT C18 Polar guard (7.5 x 2.0 mm, 1,5 µm). Temperature of the autosampler and column were kept at of 4°C and 60°C, respectively. The gradient elution was performed at 0.5 mL/min with water (0.1% formic acid (v/v)) and acetonitrile (Sigma-Aldrich). Acetonitrile increased linearly during 0 to 2.5 minutes from 5% to 15%. During 2.51-2.80 minutes the column was flushed with 100% acetonitrile. After that the gradient went back to 5% acetonitrile and remained as such until the end of the run at 3 minutes. As detection mode, multiple reaction monitoring (MRM) in negative mode was used. The parameters for detection included: curtain gas 20 psi, ion spray voltage -1200 V, temperature 700°C, ion source gas (1) 60 psi, ion source gas (2) 80 psi. Further, compound dependent parameters can be found in Supplemental table 1.

**Supplemental table 1. MRM transitions of SCFAs**

| 3-NPH derivatized Compound | Q1 mass (m/z) | Q3 mass (m/z) | Declustering potential (V) | Collision energy (V) | Collision cell exit potential (V) | Dwell time (ms) |
| --- | --- | --- | --- | --- | --- | --- |
| Acetate | 195.0 | 153.0 | -45 | -20 | -7 | 25 |
| Propionate | 208.0 | 165.0 | -125 | -18 | -9 | 25 |
| Butyrate | 222.0 | 137.0 | -135 | -26 | -9 | 25 |
| Acetate-d_3_ | 197.0 | 137.0 | -45 | -26 | -9 | 10 |
| Propionate-d_3_ | 211.0 | 137.0 | -125 | -26 | -9 | 10 |
| Butyrate-d_7_ | 229.0 | 137.0 | -135 | -26 | -9 | 10 |

**Protocol faecal sample DNA extraction and preparation for sequencing**

DNA was extracted from samples using MagPure Stool DNA KF Kit B (MAGEN, Guangzhou, China) according to manual instruction. Transfer 100-200 mg sample to the centrifuge tube with grinding beads. Add 1 mL Buffer ATL/PVP-10, grinding the sample in the grinding machine(Shanghai Jingxin Tech, China) and incubating at 65℃ for 20 min. The mixture was centrifuged at 14000 × g for 5 minutes (Eppendorf, German). Then the supernatant was transferred to a new tube. 0.6 mL Buffer PCI add to the sample and then mix thoroughly by vortexing for 15 seconds. The mixture was centrifuged at 18213 x g for 10 minutes. The supernatant was transferred to deep well plate with magnetic beads binding solution (600μL Buffer with magnetic beads +20μL Proteinase K+5μL RNase A, 700μL Wash 1, 700μL Wash 2, 700μL Wash 3, 100μL Elution Buffer. Transfer the sample to corresponding place in deep well plate of the machine (Kingfisher, Thermo Fisher, USA). Begin the corresponding program in Kingfisher. Transfer the DNA to 1.5 mL centrifuge tube for storage when the program runs out.

The library was prepared by 2 × Phanta Max Master Mix (VAZYME, China) polymerase, and the V3V4 variable region of 16S rDNA of bacteria was amplified by forward and reverse PCR degenerate primers F and R (338F:ACTCCTACGGGAGGCAGCAG, 806R:GGACTACHVGGGTWTCTAAT). PCR enrichment was performed in a 50 μL reaction containing 30ng template and fusion PCR primers. PCR cycling conditions were as follows: 95°C for 3 minutes; 30 cycles of 95°C for 15 seconds, 56°C for 15 seconds, 72°C for 45 seconds and final extension at 72°C for 5 minutes. PCR products were purified by DNA magnetic beads (BGI, LB00V60).

Next, the final double strand library products are denatured to generate the single strand library products. Then, the circularization reaction is set up to get single strand circularized DNA products. Any single strand linear DNA will be digested to remove. The final single strand circularized library is amplified with phi29 and rolling circle amplification (RCA) to generate the DNA nano ball (DNB) which carries multiple copies of the initial single stranded library molecule. The DNBs are loaded into the patterned nanoarray and sequencing reads of PE300 bases length are generated with DNBSEQ-G400 platform (BGI-Shenzhen, China).

**DATA**


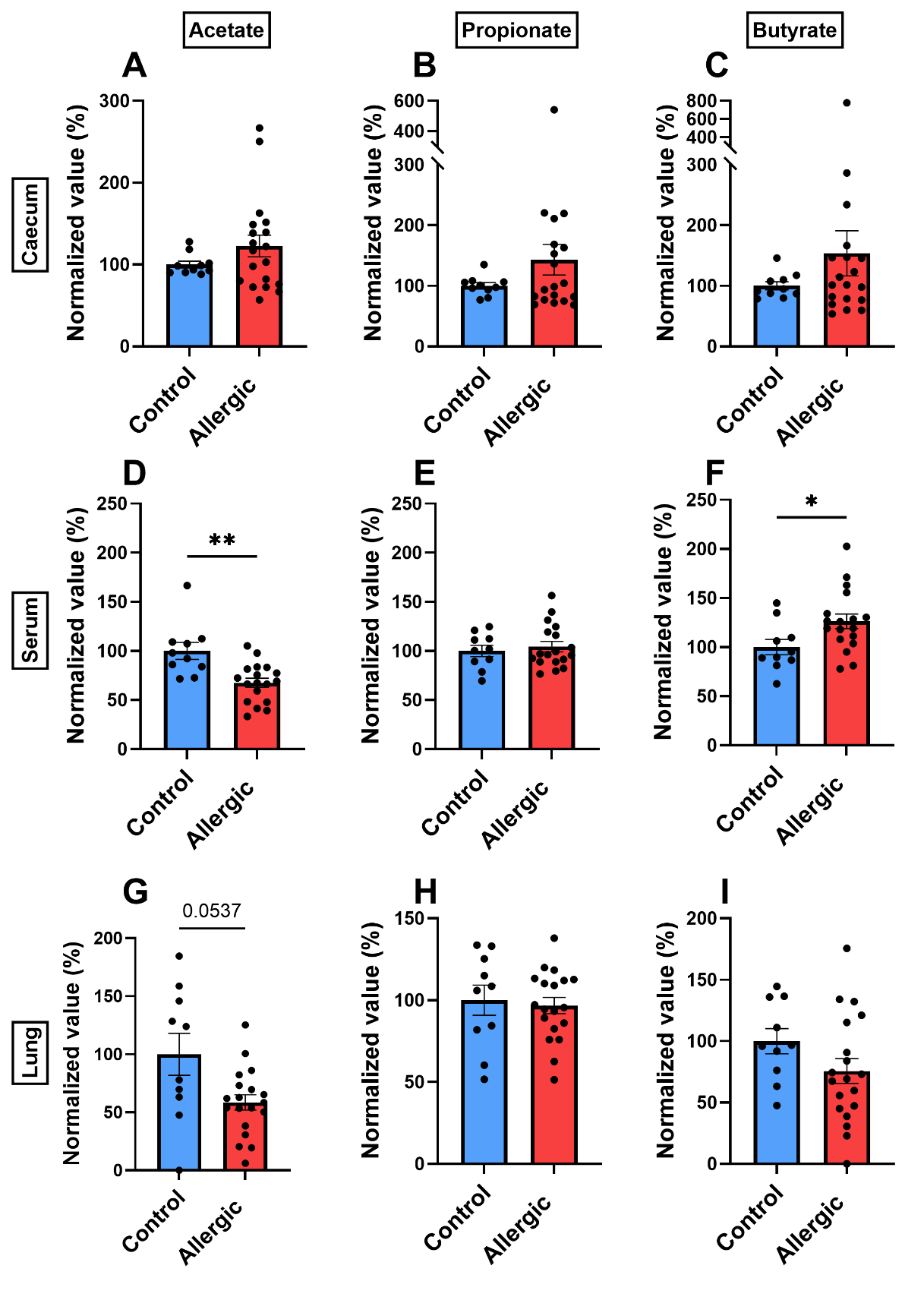


**Supplemental figure 2. Comparison of acetate, propionate and butyrate levels in different tissues (caecum content homogenate, serum, lung homogenate) between control and allergic mice.** A-C) SCFA levels in caecum, D-F) serum, or G-I) lung of control or allergic mice. Results of two independent experiments are combined in the graphs, whereby the average control values of the individual experiments were set to 100%. Values of all mice were calculated as percentage of the average control value per experiment. Data were analyzed by an unpaired t-test. Data were transformed if needed. N=10 for control. N = 20 for allergic. Data are shown as mean ± SEM. (*P<0.05, **P<0.01).
